# Micropapillary or solid component predicts worse prognosis in pathological IA stage lung adenocarcinoma: A meta-analysis

**DOI:** 10.1097/MD.0000000000036503

**Published:** 2023-12-08

**Authors:** Yifan Wang, Jingguo Hu, Yu Sun, Yusong Lu

**Affiliations:** a Department of Thoracic Surgery, Affiliated Hospital of Chengdu University, Chengdu, China.

**Keywords:** IA stage, lung adenocarcinoma, meta-analysis, micropapillary pattern, solid pattern

## Abstract

**Background:**

Micropapillary and solid patterns indicate worse survival in lung adenocarcinoma patients, even in pathological stage IB patients. However, whether the presence of micropapillary or solid components is related to worse prognosis in pathological IA stage lung adenocarcinoma remains unclear.

**Methods:**

Several databases were searched up to December 31, 2022 for relevant studies investigating the association between micropapillary and solid components and the survival of IA stage lung adenocarcinoma patients. Primary and secondary outcomes are disease-free survival (DFS) and overall survival (OS), respectively. Hazard ratios (HRs) and 95% confident intervals (CIs) were combined, and subgroup analysis stratified by the pathological subtype and proportion of components was further performed.

**Results:**

A total of 19 studies with 12,562 cases were included. Pooled results indicated that micropapillary or solid components obviously predicted worse DFS (HR = 2.40, 95% CI: 1.96–2.94, *P* < .001) and OS (HR = 2.30, 95% CI: 1.68–3.15, *P* < .001). Subgroup analysis based on pathological subtype showed that both micropapillary and solid components were significantly associated with worse DFS (micropapillary: HR = 2.70, 95% CI: 1.70–4.28, *P* < .001; solid: HR = 3.98, 95% CI: 2.10–7.54, *P* < .001) and OS (micropapillary: HR = 2.29, 95% CI: 1.17–4.48, *P* = .015; solid: HR = 4.18, 95% CI: 1.72–10.17, *P* = .002). In addition, further subgroup analysis stratified by the proportion of micropapillary and solid components (>5%/1% or predominant) showed similar results.

**Conclusion:**

Micropapillary and solid patterns predicted a significantly worse prognosis in pathological IA stage lung adenocarcinoma patients.

## 1. Introduction

Lung cancer remains the leading cause of tumor-related deaths worldwide, and adenocarcinoma accounts for the most common subtype of lung cancer, especially in early-stage cases.^[1,2]^ According to the novel classification described by the International Association for the Study of Lung Cancer (IASLC)/American Thoracic Society (ATS)/European Respiratory Society (ERS) in 2011, invasive adenocarcinoma is divided into the following histopathological subtypes: lepidic-, papillary-, acinar-, micropapillary and solid predominant adenocarcinoma.^[3]^ Afterward, this novel pathological typing system has verified that the predominant subtype could relatively predict the prognosis of invasive lung adenocarcinoma, and it is widely reported that micropapillary and solid predominant subtypes are significantly related to poor prognosis.^[4–7]^ In addition, many studies have indicated that even a small proportion of micropapillary and solid components also have an obvious impact on patient survival.^[8–11]^ There are already several meta-analyses that have verified that a small proportion of micropapillary or solid components are associated with poor prognosis in lung adenocarcinoma patients.^[12,13]^ However, none of them focused on IA stage cases.

For pathological IA stage lung adenocarcinoma, surgical resection is the main treatment, and the prognosis for most patients is fairly good.^[14,15]^ Unfortunately, a small percentage of IA stage patients, approximately 5%, with risk factors such as spread through air space and elevated inflammation index experience recurrence or metastasis after surgery.^[16]^ Overall, postoperative adjuvant treatment is not recommended for pathological IA lung adenocarcinoma patients. However, for pathological IB stage lung adenocarcinoma, it has been identified that a solid or micropapillary pattern is significantly associated with the survival of IB stage lung adenocarcinoma patients, and patients with a solid/micropapillary pattern benefit from postoperative chemotherapy.^[13,17,18]^ Thus, it is necessary to clarify the predictive role of a micropapillary/solid component for survival among pIA stage lung adenocarcinoma patients, which contributes to the accurate prediction of postoperative prognosis and formulation of therapeutic strategies.

Therefore, the current meta-analysis aimed to further determine the association between micropapillary or solid components and the prognosis of pathological IA stage lung adenocarcinoma patients based on current relevant evidence.

## 2. Materials and methods

This meta-analysis was conducted according to the Preferred Reporting Items for Systematic Reviews and Meta-Analyses guidelines (2020).^[19]^

### 2.1. Literature search

The Medline, EMBASE, Web of Science and CNKI electronic databases were searched from inception to December 31, 2022. The following key words were used during the search: lung, pulmonary, adenocarcinoma; micropapillary, solid, IA stage, survival, prognostic and prognosis. The detailed search strategy was as follows: (lung OR pulmonary) AND (micropapillary OR solid) AND adenocarcinoma AND IA stage AND (survival OR prognostic OR prognosis). The free words and MeSH terms were applied, and references of included studies were also reviewed.

### 2.2. Inclusion and exclusion criteria

The inclusion criteria were as follows: patients were diagnosed with primary IA stage lung adenocarcinoma pathologically; patients received resection; the disease-free survival (DFS) or (and) overall survival (OS) of patients with and without micropapillary or solid component was (were) compared; and hazard ratios (HRs) and 95% confidence intervals (CIs) for DFS and OS were reported in articles or survival curves were provided.

The exclusion criteria were as follows: insufficient data to assess the association between micropapillary or solid components and DFS or OS; duplicated or overlapping data; and meeting abstracts, letters, editorials, reviews or animal trials.

### 2.3. Data extraction

The following information was collected from each included meta-analysis: the first author, publication year, country, sample size, cutoff value of micropapillary or solid component, observation factor (micropapillary pattern or solid pattern), endpoint and Newcastle–Ottawa Scale (NOS) score.

### 2.4. Methodological quality assessment

All included studies were retrospective, and the NOS scoring system was used to assess the methodological quality of each study. Studies with an NOS score ≥ 6 were defined as high-quality studies.^[20]^

The literature retrieval, selection, data extraction and quality assessment were all independently conducted by 2 authors, and any disagreements were resolved by team discussion.

### 2.5. Statistical analysis

All statistical analyses were conducted by STATA 12.0 software. HRs with 95% CIs were combined to identify the association between the presence of micropapillary or solid components and the survival of IA stage patients. If HRs with 95% CIs were not reported in articles, then they would be calculated according to Kaplan–Meier survival curves using the method described by Tierney et al^[21]^ The heterogeneity between included studies was assessed by *I*^2^ statistics and Q tests. If significant heterogeneity was observed as *I*^2^ greater than 50% or a *P* value less than .1, the random-effects model was used; otherwise, the fixed-effects model was used.^[22]^ Subgroup analysis based on pathological subtype (micropapillary or solid pattern) and proportion of micropapillary and solid components was further conducted. In addition, sensitivity analysis was conducted to identify the source of heterogeneity and evaluate the stability of the pooled results. Furthermore, Begg’s funnel plot was applied to detect potential publication bias.^[23]^

## 3. Results

### 3.1. Literature search process

**Figure 1.**
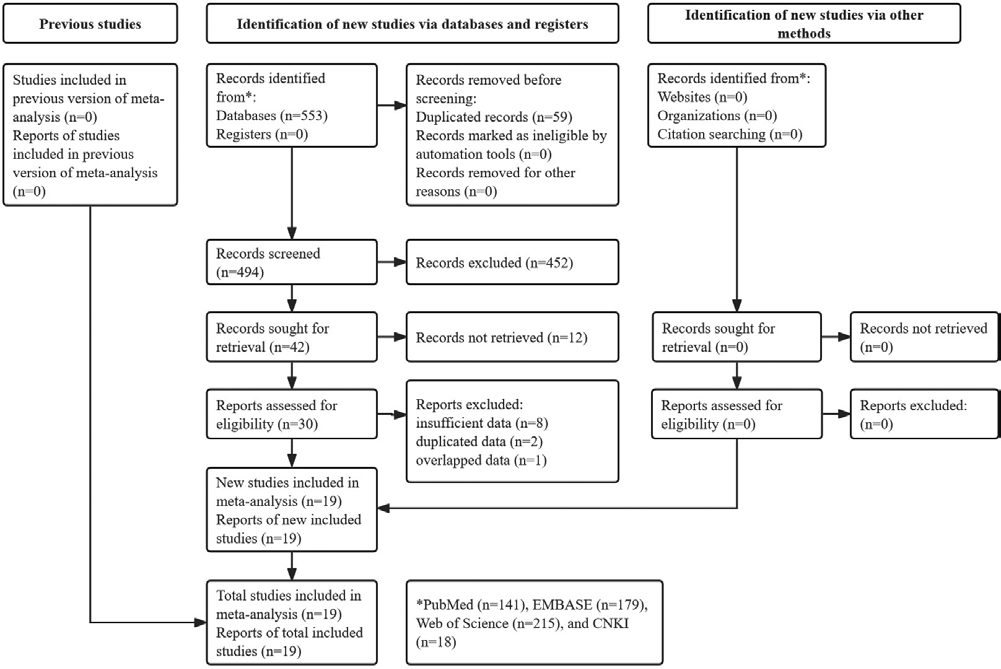
Prisma flow diagram.

The specific literature selection process is shown in Figure 1. A total of 19 studies were eventually included in this meta-analysis.^[24–42]^

### 3.2. Basic characteristics of the included studies

Most included studies were retrospective and from Asian countries, including China, Japan and the Republic of Korea, with a total of 12,562 participants. The sample size ranged from 77 to 2504. Notably, the study by Chen D et al analyzed 2 separate populations; therefore, we identified them as 2 studies during the following analysis. In addition, all studies were high-quality studies with an NOS score of 6 or higher. The other detailed characteristics are presented in Table 1.

**Table 1:** Basic characteristics of included studies.

Author	Year	Country	Sample size	Cutoff value of MIP component	Cutoff value of SD component	Observation factor	Endpoint	Source of HR	NOS
Sumiyoshi^[24]^	2013	Japan	256	>5%	-	MIP+	DFS, OS	E	7
Hirano^[25]^	2014	Japan	218	>5%	-	MIP+	OS	E	7
Luo^[26]^	2017	China	2504	-	Predominant	SD+	DFS, OS	R	7
Wang^[27]^	2018	China	1965	>5%	>5%	MIP+/SD+	DFS, OS	R	6
Chen C^[28]^	2020	China	77	>5%	>5%	MIP+, SD+	OS	E	6
Chen D^[29]^	2020	China	1543	Predominant	-	MIP+	DFS, OS	E	6
Chen D^[29]^	2020	China	714	Predominant	-	MIP+	DFS, OS	E	6
Zhang^[30]^	2020	China	90	Predominant	Predominant	MIP+/SD+	OS	R	6
Peng^[31]^	2021	China	422	>5%	>5%	MIP+/SD+, MIP+, SD+	DFS, OS	R/E	7
Choi^[33]^	2021	Korea	299	0-5%	0-5%	MIP+/SD+	DFS	R	6
Jeon^[34]^	2021	Republic of Korea	429	>20%	>20%	MIP+/SD+	DFS, OS	R	6
Zhai^[35]^	2021	China	484	>5%	>5%	MIP+/SD+	DFS, OS	R	7
Chen Y^[32]^	2021	China	314	>5%	>5%	MIP+, SD+	DFS, OS	R	7
Zhang^[36]^	2021	China	435	-	-	MIP+, SD+	DFS, OS	R	7
Bertoglio^[37]^	2022	Italy	326	>5%	>5%	MIP+/SD+	DFS, OS	E	7
Chen C^[38]^	2022	China	814	>1%	>1%	MIP+, SD+	DFS	R	6
Huang^[39]^	2022	China	595	>10%	>10%	MIP+/SD+	DFS, OS	R	7
Jin^[40]^	2022	China	321	>5%	>5%	MIP+/SD+	DFS	R	7
Okubo^[42]^	2022	Japan	380	Predominant	Predominant	MIP+, SD+	DFS	R	6
Lu^[41]^	2022	China	306	-	-	MIP+/SD+	DFS, OS	R	7

DFS = disease-free survival, E = estimated, HR = hazard ratio, MIP = micropapillary, NOS = Newcastle-Ottawa Scale, NR = not reported, OS = overall survival, R = reported, SD = solid.

### 3.3. The relationship between micropapillary or solid components and survival

**Figure 2.**
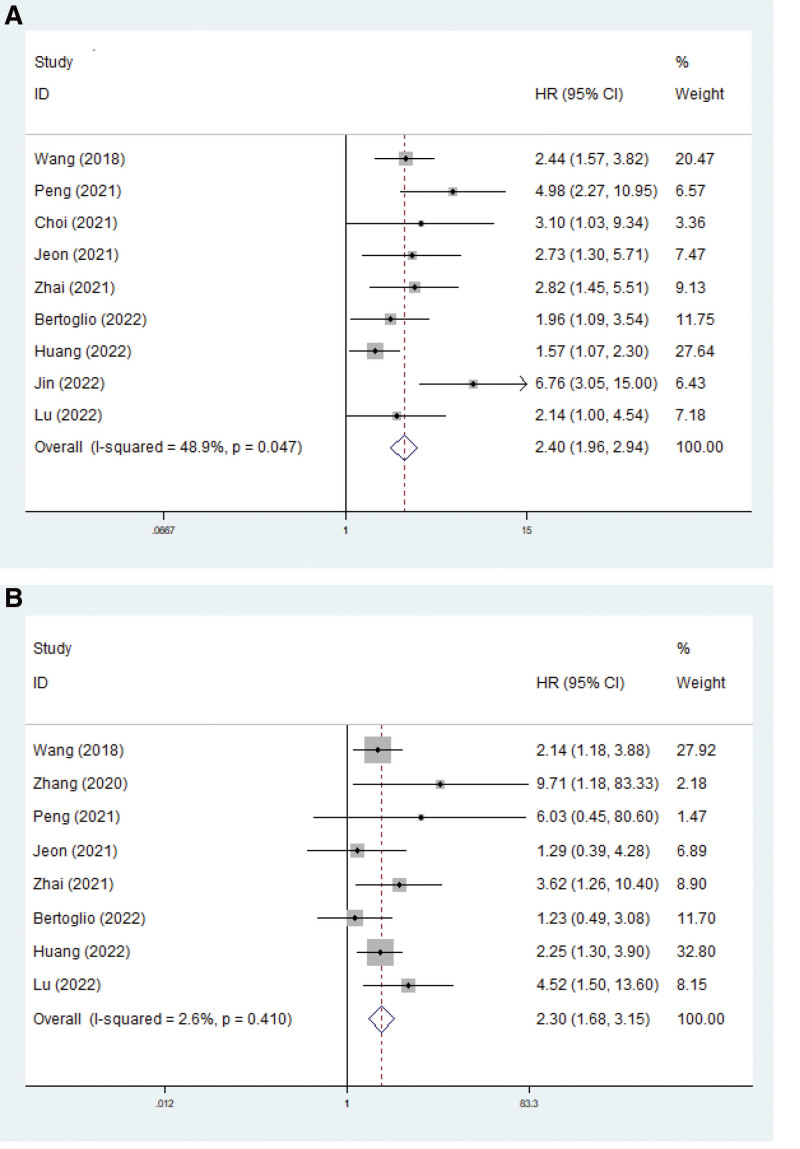
The association between micropapillary or solid component and disease-free survival (A) and overall survival (B) in pathological IA stage lung adenocarcinoma patients.

Nine and 8 studies explored the predictive role of micropapillary or solid components for DFS and OS, respectively. The pooled results demonstrated that micropapillary or solid components predicted worse DFS (HR = 2.40, 95% CI: 1.96–2.94, *P* < .001; *I*^2^ = 48.9%, *P* = .047) (Fig. 2A) and OS (HR = 2.30, 95% CI: 1.68–3.15, *P* < .001; *I*^2^ = 2.6%, *P* = .410) (Fig. 2B; Table 2).

**Table 2:** Results of meta-analysis.

	No. of studies	Hazard ratio	95% confidence interval	*P* value	*I*^2^ (%)	*P* value
MIP or SD component						
Disease-free survival	9	2.40	1.96–2.94	<.001	48.9	.047
Overall survival	8	2.30	1.68–3.15	<.001	2.6	.410
MIP component						
Disease-free survival	7	2.70	1.70–4.28	<.001	66.3	.004
Predominant	2	1.61	1.20–2.16	.001	73.8	.022
>5%/1%	4	3.19	2.04–4.99	<.001	0.0	.535
Overall survival	6	2.29	1.17–4.48	.015	78.8	<.001
Predominant	1	1.37	0.95–3.22	.226	58.6	.120
>5%	4	2.56	0.65–10.02	.177	79.0	.003
SD component						
Disease-free survival	6	3.98	2.10–7.54	<.001	72.8	.003
Predominant	2	6.44	0.88–46.91	.066	83.0	.015
>5%	3	4.43	1.50–13.07	.007	79.9	.007
Overall survival	5	4.18	1.72–10.17	.002	66.4	.018
Predominant	1	2.36	0.97–5.71	.058	–	–
>5%	3	7.30	1.36–39.21	.021	74.1	.021

MIP = micropapillary, SD = solid.

### 3.4. The relationship between micropapillary component and survival

**Figure 3.**
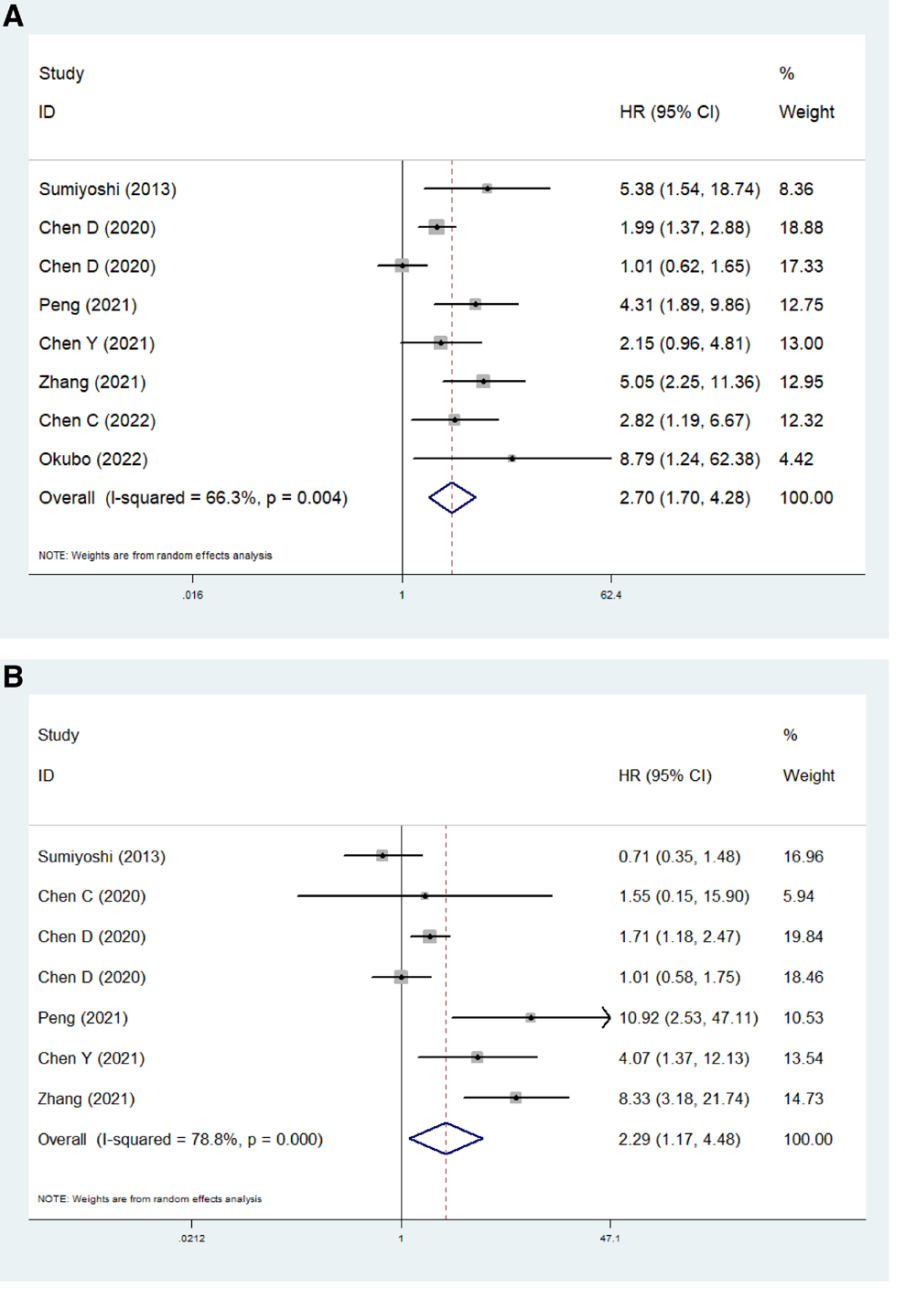
The association between micropapillary component and disease-free survival (A) and overall survival (B) in pathological IA stage lung adenocarcinoma patients.

Seven and 6 studies explored the relationship between the micropapillary component and DFS and OS in IA stage lung adenocarcinoma. The pooled results showed that the presence of a micropapillary component indicated significantly worse DFS (HR = 2.70, 95% CI: 1.70–4.28, *P* < .001; *I*^2^ = 66.3%, *P* = .004) (Fig. 3A) and OS (HR = 2.29, 95% CI: 1.17–4.48, *P* = .015; *I*^2^ = 78.8%, *P* < .001) (Fig. 3B). Furthermore, subgroup analysis based on the proportion of micropapillary component (predominant or > 5%/1%) showed similar results, which further identified that the presence of micropapillary component was obviously related to worse survival of pathological IA stage lung adenocarcinoma patients regardless of the proportion (Table 2).

### 3.5. The association between solid component and survival

**Figure 4.**
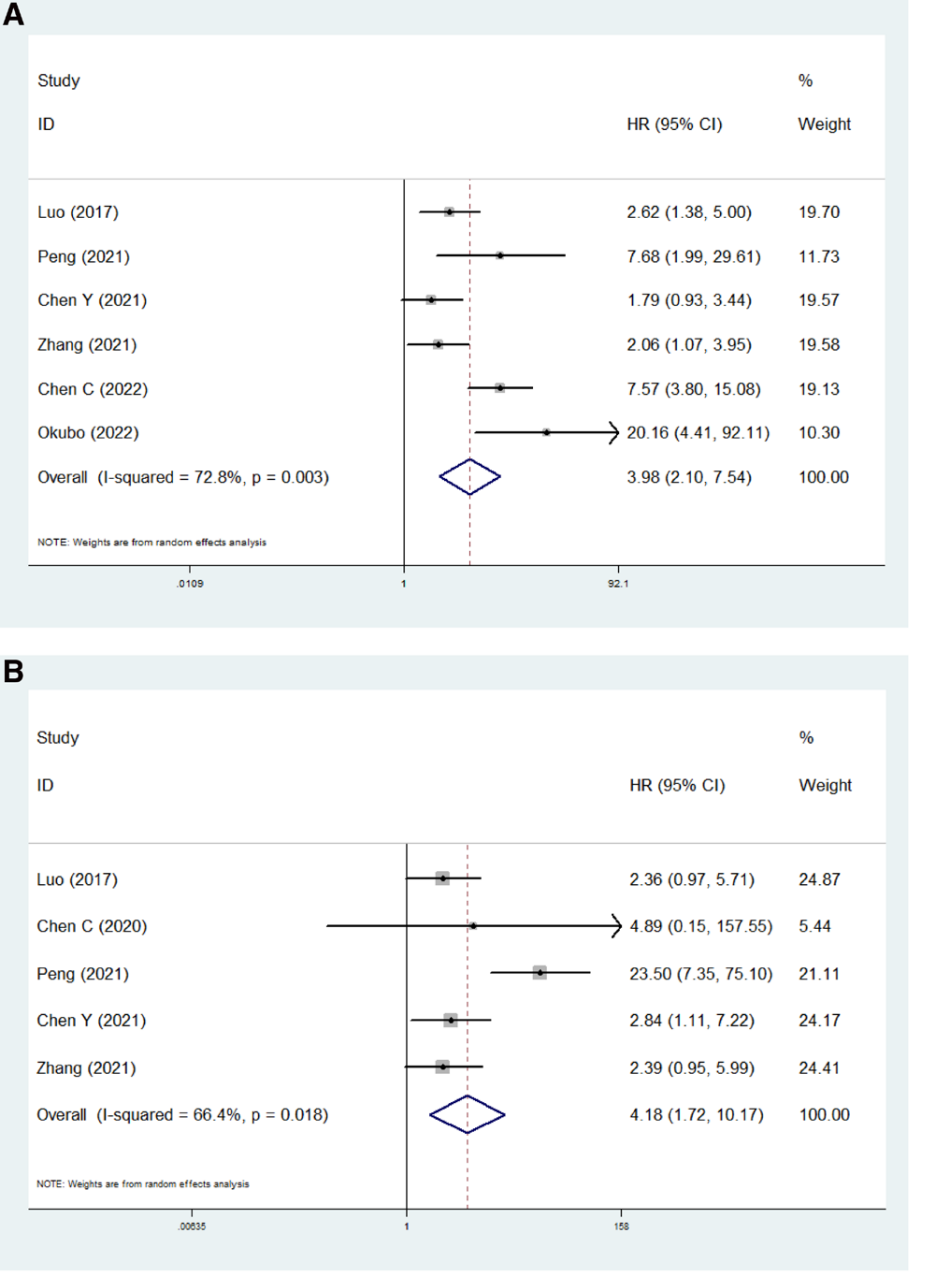
The association between solid component and disease-free survival (A) and overall survival (B) in pathological IA stage lung adenocarcinoma patients.

Six and 5 included studies explored the association of the solid component with DFS^[26,31,32,36,38,42]^ and OS^[26,28,31,32,36]^ in IA stage lung adenocarcinoma, respectively. The pooled results demonstrated that the presence of a solid component was significantly associated with shortened DFS (HR = 3.98, 95% CI: 2.10–7.54, *P* < .001; *I*^2^ = 72.8%, *P* = .003) (Fig. 4A) and OS (HR = 4.18, 95% CI: 1.72–10.17, *P* = .002; *I*^2^ = 66.4%, *P* = .018) (Fig. 4B). Similarly, subgroup analysis stratified by the proportion of solid component (predominant or > 5%) was also conducted, which indicated that the presence of solid component was associated with worse prognosis in pathological IA stage lung adenocarcinoma patients regardless of the proportion, although some results did not reach statistical significance (Table 2).

### 3.6. Sensitivity analysis and publication bias

Sensitivity analysis was performed on the association of micropapillary or solid components (Figure S1A and B, Supplemental Digital Content, http://links.lww.com/MD/K958 for DFS and OS), micropapillary components (Figure S2A and B, Supplemental Digital Content, http://links.lww.com/MD/K959 for DFS and OS) and solid components (Figure S3A and B, Supplemental Digital Content, http://links.lww.com/MD/K960 for DFS and OS) with the survival of IA stage lung adenocarcinoma patients. Overall, the sensitivity analysis revealed that the results of this meta-analysis were stable and reliable.

Begg’s funnel plots were constructed to identify publication bias regarding the association of micropapillary or solid components (Figures S1C and D, Supplemental Digital Content, http://links.lww.com/MD/K958 for DFS and OS), micropapillary components (Figures S2C and D, Supplemental Digital Content, http://links.lww.com/MD/K959 for DFS and OS) and solid components (Figures S3C and D, Supplemental Digital Content, http://links.lww.com/MD/K960 for DFS and OS) with the survival of IA stage lung adenocarcinoma patients. Overall, no obvious publication bias was observed.

## 4. Discussion

The current meta-analysis demonstrated that the micropapillary and solid components both predicted reduced survival of pathological IA stage lung adenocarcinoma patients and that the proportion of components did not cause an obvious impact on the overall results. Therefore, the presence of a micropapillary or solid component was significantly associated with poor prognosis and could serve as a reliable and important prognostic factor in IA stage lung adenocarcinoma. However, more prospective high-quality studies are still needed to further verify our findings.

The prognostic values of micropapillary and solid patterns in lung adenocarcinoma have been widely explored and reported. Pyo et al analyzed 19,502 lung adenocarcinoma cases from 48 studies and demonstrated that the micropapillary pattern was related to higher rates of lymphatic invasion (HR = 0.526), OS (HR = 1.704) and DFS (HR = 2.082).^[43]^ In addition, in the meta-analysis by Wang et al, both the presence of a micropapillary component (DFS: HR = 1.80; OS: HR = 2.26) and micropapillary predominant subtype (DFS: HR = 1.62; OS: HR = 1.53) predicted reduced survival after lung adenocarcinoma resection.^[12]^ Similarly, Miyahara et al^[44]^ included 14 eligible studies involving 12,137 operated lung adenocarcinoma patients and indicated that the solid subtype was associated with obviously worse postoperative DFS (HR = 1.26) and OS (HR = 1.51). Notably, this was the first meta-analysis that identified the predictive role of micropapillary and solid components for poor prognosis in pathological IA stage lung adenocarcinoma.

In the last few years, the relationship between solid and micropapillary patterns and adjuvant therapy has been gradually explored. Overall, patients with a micropapillary or solid component could benefit from adjuvant treatment, especially chemotherapy.^[17,18,45]^ For early-stage cases, Xu et al^[13]^ reviewed 6 studies including 956 patients and found that postoperative chemotherapy could significantly improve OS (HR = 0.58, *P* < .001) and DFS (HR = 0.51, *P* < .001) of IB stage patients with the micropapillary or solid pattern. However, few studies have explored the clinical significance of adjuvant therapy in resected IA stage lung adenocarcinoma because adjuvant therapy is not recommended for IA stage lung adenocarcinoma patients overall.

There are still much to investigate about the clinical role of micropapillary and solid patterns in pathological IA stage lung adenocarcinoma. First, as mentioned above, the relationship between adjuvant therapy and micropapillary and solid patterns remains unclear. In other words, it is necessary to identify whether the presence of a micropapillary or solid component could be regarded as an indicator to guide postoperative adjuvant therapy. Second, in most included studies, the detailed proportions of micropapillary or solid components were not analyzed, and we believe that the specific proportion has a significant impact on the prognostic value of micropapillary and solid components in IA stage lung adenocarcinoma. Third, the combination of micropapillary and solid components is common in clinics. Thus, the combination of these 2 subtypes may predict worse survival than a single subtype, which should be verified in future studies. Fourth, it is also important to explore the association between the presence of micropapillary or solid components in frozen rapid pathological examination and the procedure of pulmonary resection in IA stage lung adenocarcinoma. In other words, micropapillary and solid patterns might affect the excision extension of IA stage lung adenocarcinoma patients. Furthermore, the prognostic role of the second predominant pattern in pIA stage lung adenocarcinoma was not explored in our included studies. It is also necessary to identify the predictive role of the second predominant pattern in pIA lung adenocarcinoma.

There are several limitations in this meta-analysis. First, most included studies are retrospective and from Asian countries, which might cause some bias and limit the generality of our results. Second, due to the lack of original data, we were unable to conduct more subgroup analyses based on other important parameters, such as the T stage and differentiation grade. Third, it was not possible to conduct detailed prognostic risk analysis according to the specific proportion of micropapillary and solid components. Fourth, data about further treatment and outcomes among pIA lung adenocarcinoma patients who experienced recurrence after the operation were not reported in the included studies. Fifth, only the prognostic role of micropapillary and/or solid patterns in pIA lung adenocarcinoma was identified in this meta-analysis. In other words, the predictive role for long-term survival of other patterns might be further investigated in future studies.

## 5. Conclusion

Micropapillary and solid components both predicted significantly worse prognosis in pathological IA stage lung adenocarcinoma patients. However, more prospective high-quality studies are still needed to further verify our findings.

## Author contributions

**Conceptualization:** Yifan Wang.

**Data curation:** Jingguo Hu, Yusong Lu.

**Formal analysis:** Yifan Wang, Jingguo Hu, Yusong Lu.

**Investigation:** Yusong Lu.

**Methodology:** Yu Sun.

**Software:** Yu Sun.

**Writing – original draft:** Yifan Wang, Yu Sun.

**Writing – review & editing:** Jingguo Hu.

## Supplementary Material






