# Supplementary material for: Micropapillary or solid component predicts worse prognosis in pathological IA stage lung adenocarcinoma: A meta-analysis
Source: Medicine (Baltimore). 2023 Dec 8;102(49):e36503. doi: 10.1097/MD.0000000000036503 (PMC10713195; doi:10.1097/MD.0000000000036503)
Supplement: Supplementary file 1 [file medi-102-e36503-s001.pdf]

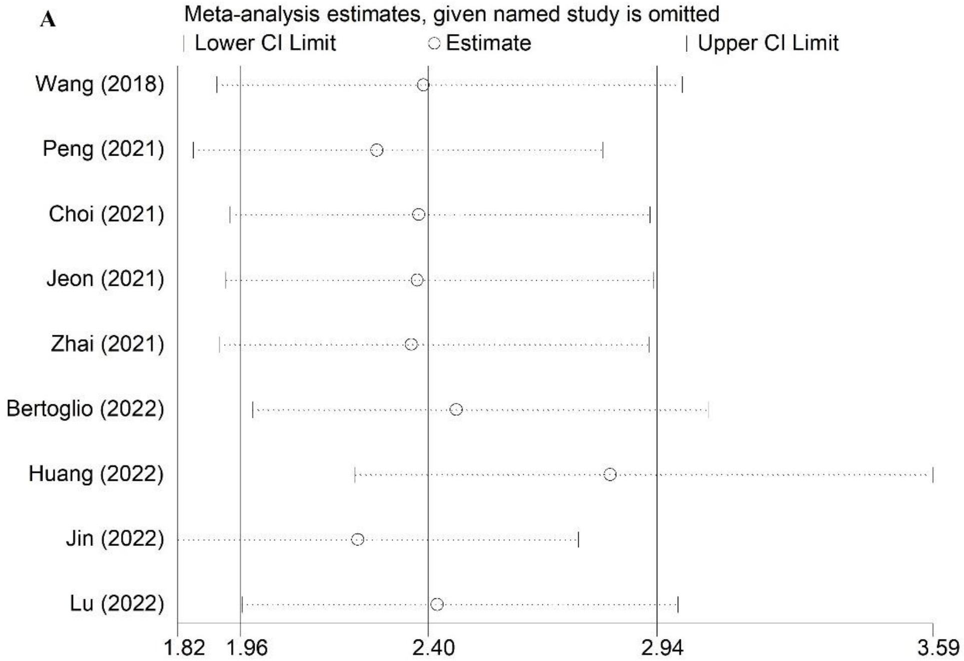
A
Meta-analysis estimates, given named study is omitted
Lower CI Limit
Estimate
Upper CI Limit
Wang (2018)
Peng (2021)
Choi (2021)
Jeon (2021)
Zhai (2021)
Bertoglio (2022)
Huang (2022)
Jin (2022)
Lu (2022)
1.82
1.96
2.40
2.94
3.59

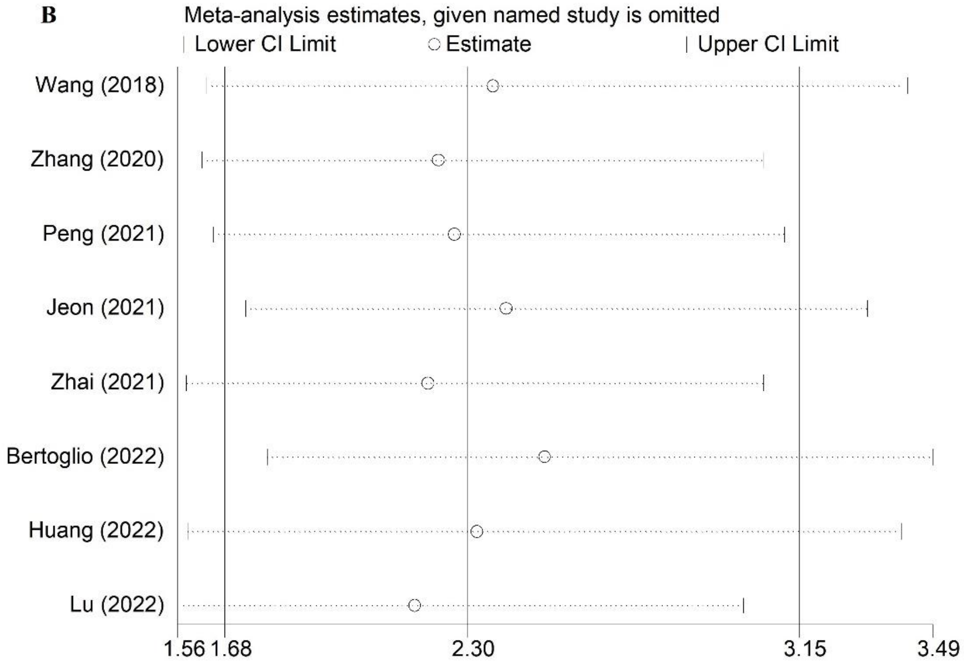
B
Meta-analysis estimates, given named study is omitted
Lower CI Limit
Estimate
Upper CI Limit
Wang (2018)
Zhang (2020)
Peng (2021)
Jeon (2021)
Zhai (2021)
Bertoglio (2022)
Huang (2022)
Lu (2022)
1.56
1.68
2.30
3.15
3.49

C

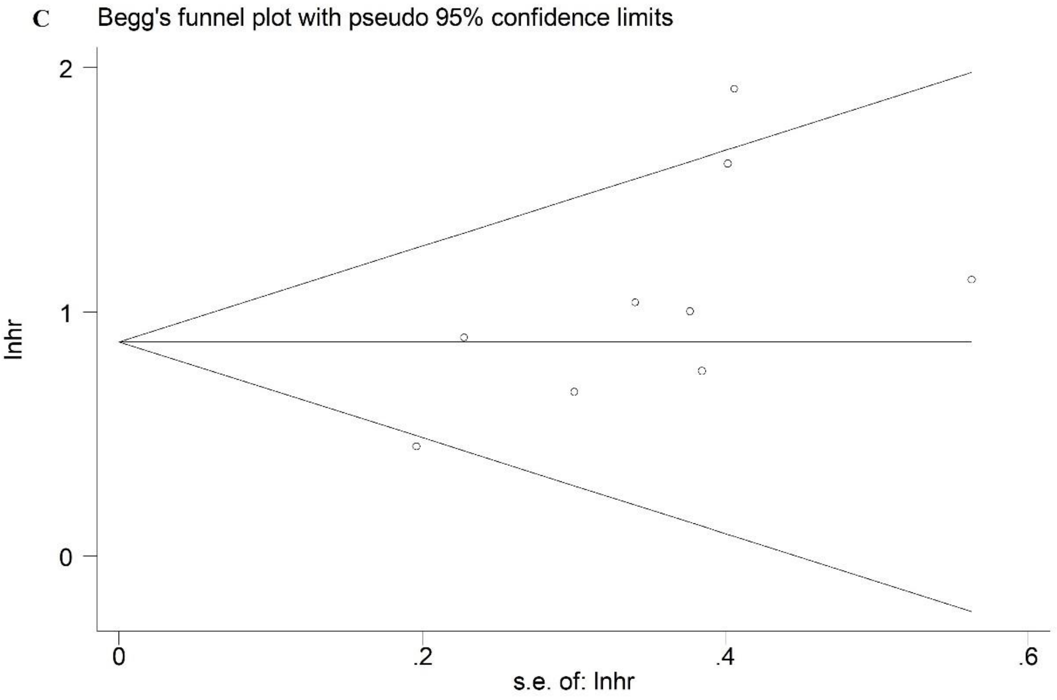
Begg's funnel plot with pseudo 95% confidence limits
2
1
0
lnhr
0
.2
.4
.6
s.e. of: lnhr

D

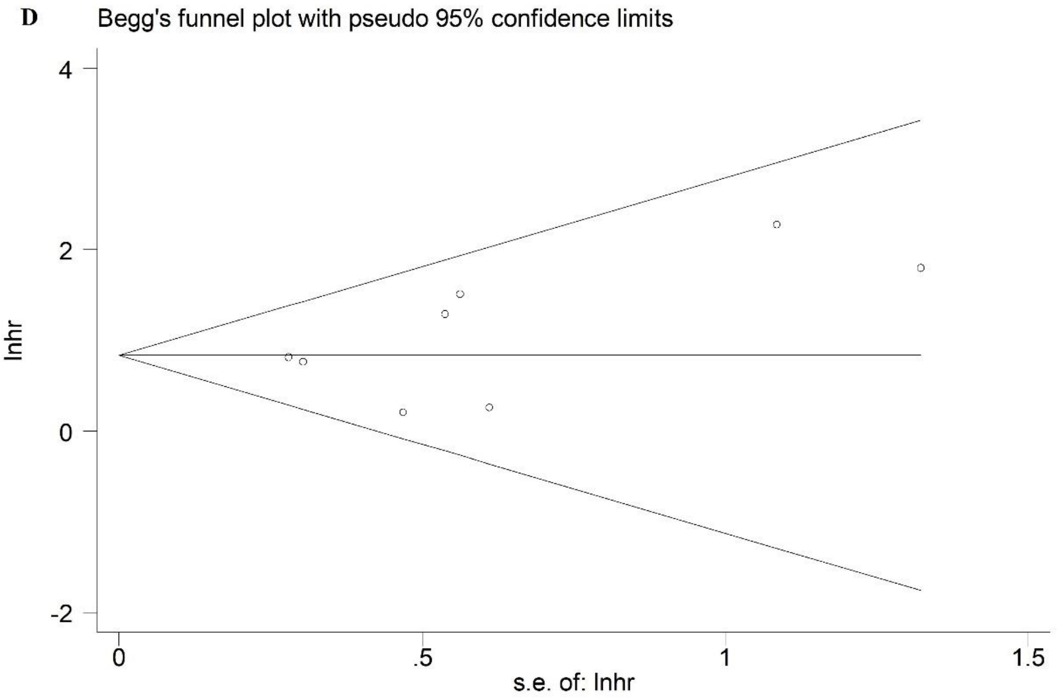
Begg's funnel plot with pseudo 95% confidence limits
4
2
0
-2
lnhr
0
.5
1
1.5
s.e. of: lnhr

Supplementary figure 1. Sensitivity analysis for the association between micropapillary or solid component and disease-free survival (A) and overall survival (B) in pathological IA stage lung adenocarcinoma patients. Begg's funnel plot for the association between micropapillary or solid component and disease-free survival (C) and overall survival (D) in pathological IA stage lung adenocarcinoma patients.
