# Supplementary material for: Micropapillary or solid component predicts worse prognosis in pathological IA stage lung adenocarcinoma: A meta-analysis
Source: Medicine (Baltimore). 2023 Dec 8;102(49):e36503. doi: 10.1097/MD.0000000000036503 (PMC10713195; doi:10.1097/MD.0000000000036503)
Supplement: Supplementary file 2 [file medi-102-e36503-s002.pdf]

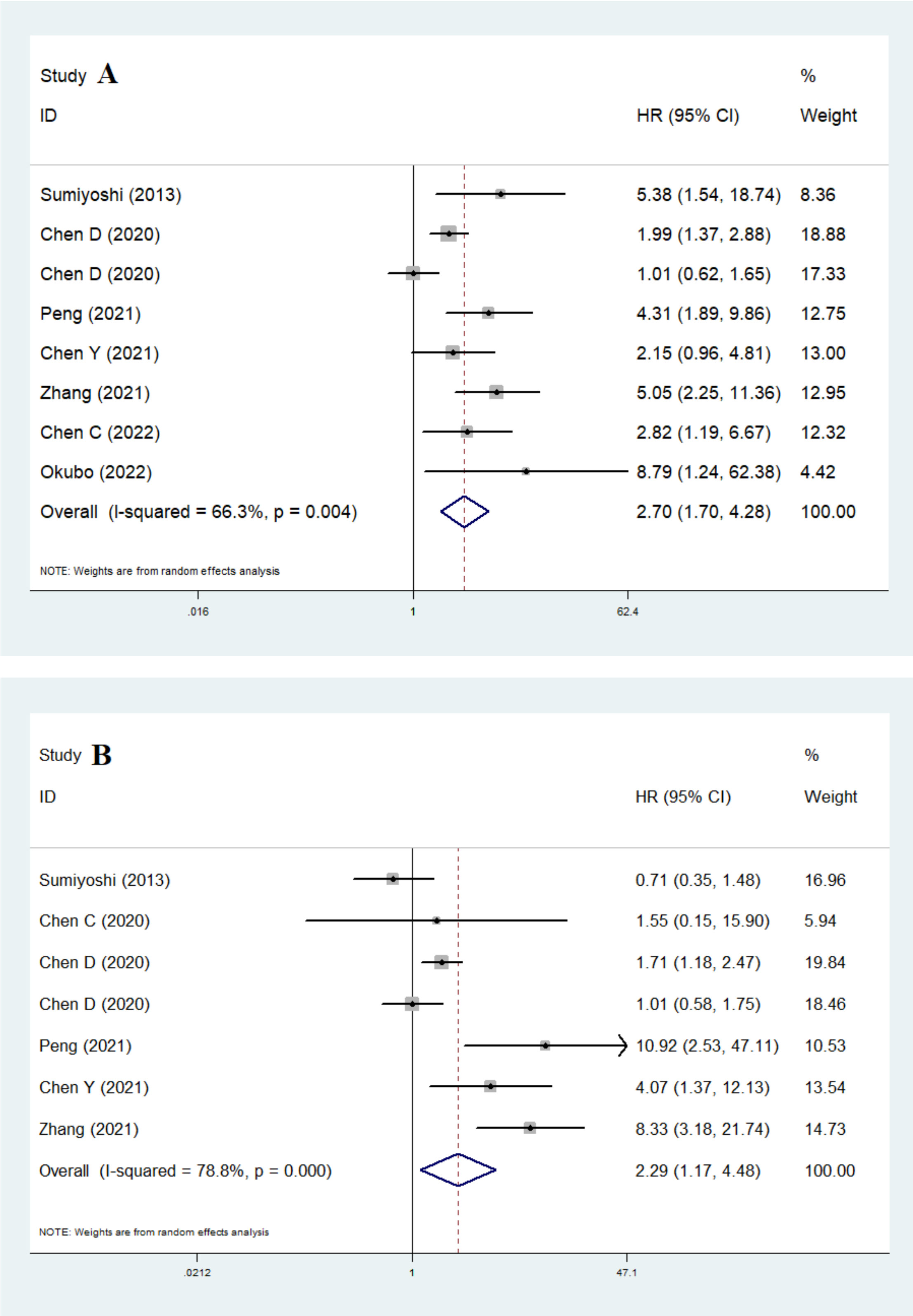
Study A
%
ID
HR (95% CI)
Weight
Sumiyoshi (2013) 5.38 (1.54, 18.74) 8.36
Chen D (2020) 1.99 (1.37, 2.88) 18.88
Chen D (2020) 1.01 (0.62, 1.65) 17.33
Peng (2021) 4.31 (1.89, 9.86) 12.75
Chen Y (2021) 2.15 (0.96, 4.81) 13.00
Zhang (2021) 5.05 (2.25, 11.36) 12.95
Chen C (2022) 2.82 (1.19, 6.67) 12.32
Okubo (2022) 8.79 (1.24, 62.38) 4.42
Overall (I-squared = 66.3%, p = 0.004) 2.70 (1.70, 4.28) 100.00
NOTE: Weights are from random effects analysis
.016
1
62.4
Study B
%
ID
HR (95% CI)
Weight
Sumiyoshi (2013) 0.71 (0.35, 1.48) 16.96
Chen C (2020) 1.55 (0.15, 15.90) 5.94
Chen D (2020) 1.71 (1.18, 2.47) 19.84
Chen D (2020) 1.01 (0.58, 1.75) 18.46
Peng (2021) 10.92 (2.53, 47.11) 10.53
Chen Y (2021) 4.07 (1.37, 12.13) 13.54
Zhang (2021) 8.33 (3.18, 21.74) 14.73
Overall (I-squared = 78.8%, p = 0.000) 2.29 (1.17, 4.48) 100.00
NOTE: Weights are from random effects analysis
.0212
1
47.1

C

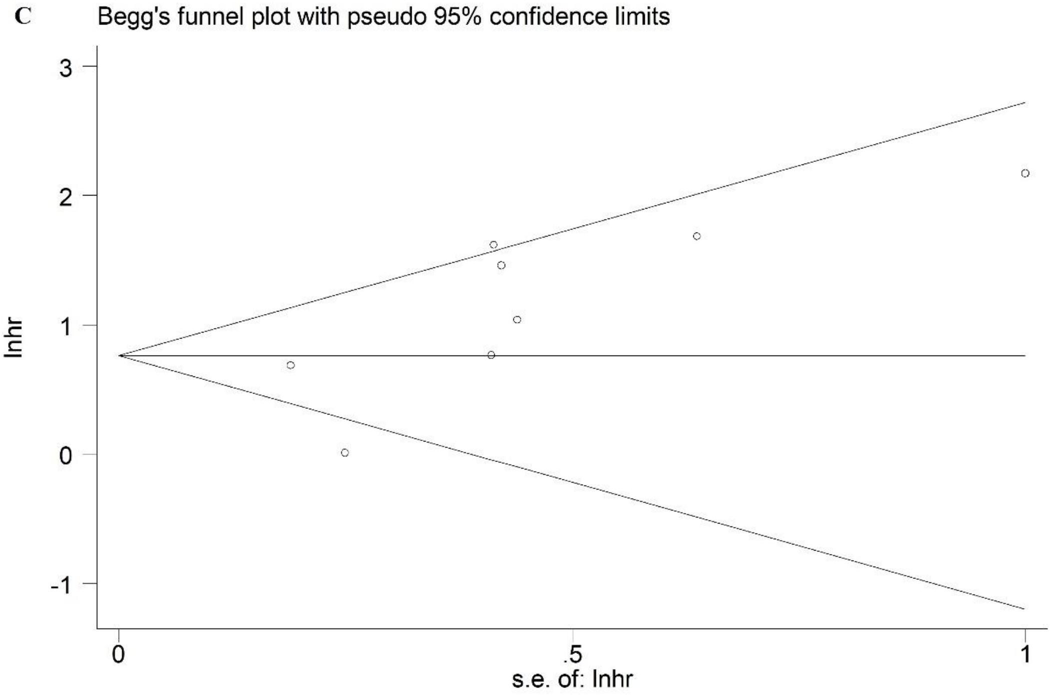

D

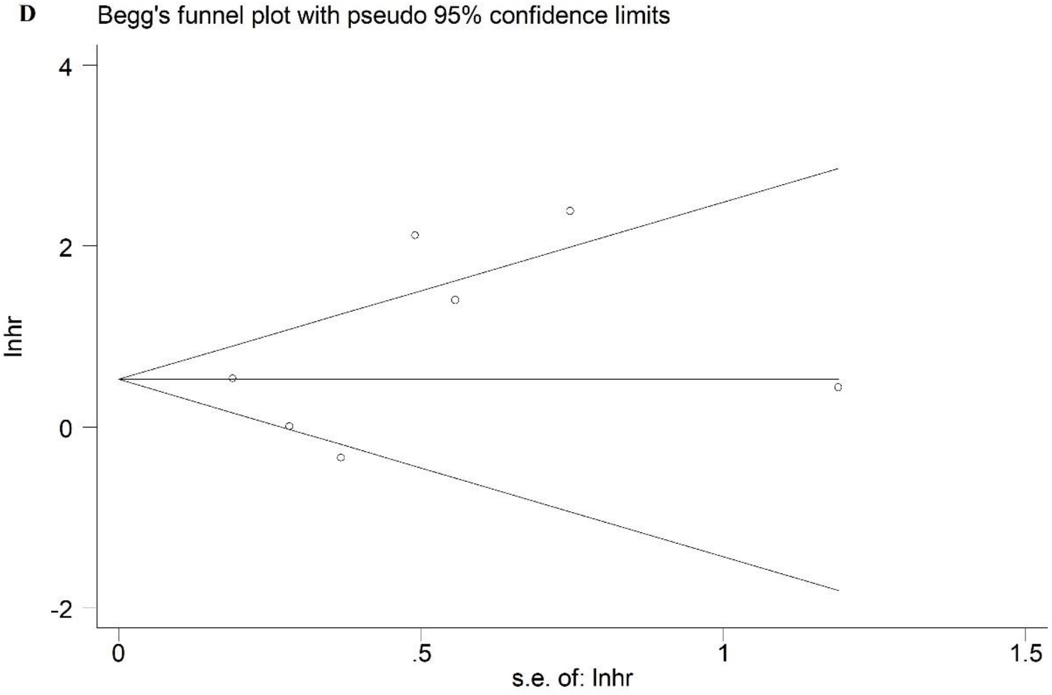

Supplementary figure 2. Sensitivity analysis for the association between micropapillary component and disease-free survival (A) and overall survival (B) in pathological IA stage lung adenocarcinoma patients. Begg's funnel plot for the association between micropapillary component and disease-free survival (C) and overall survival (D) in pathological IA stage lung adenocarcinoma patients.
