# Supplementary material for: Micropapillary or solid component predicts worse prognosis in pathological IA stage lung adenocarcinoma: A meta-analysis
Source: Medicine (Baltimore). 2023 Dec 8;102(49):e36503. doi: 10.1097/MD.0000000000036503 (PMC10713195; doi:10.1097/MD.0000000000036503)
Supplement: Supplementary file 3 [file medi-102-e36503-s003.pdf]

A

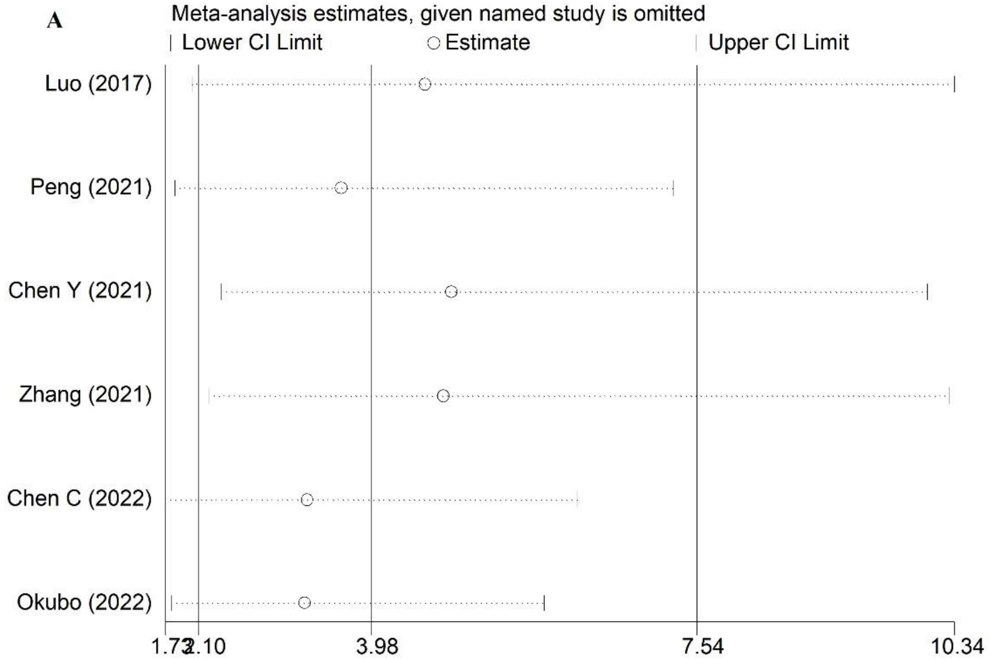
Meta-analysis estimates, given named study is omitted
Lower CI Limit
Estimate
Upper CI Limit
Luo (2017)
Peng (2021)
Chen Y (2021)
Zhang (2021)
Chen C (2022)
Okubo (2022)
2.10
3.98
7.54
10.34

B

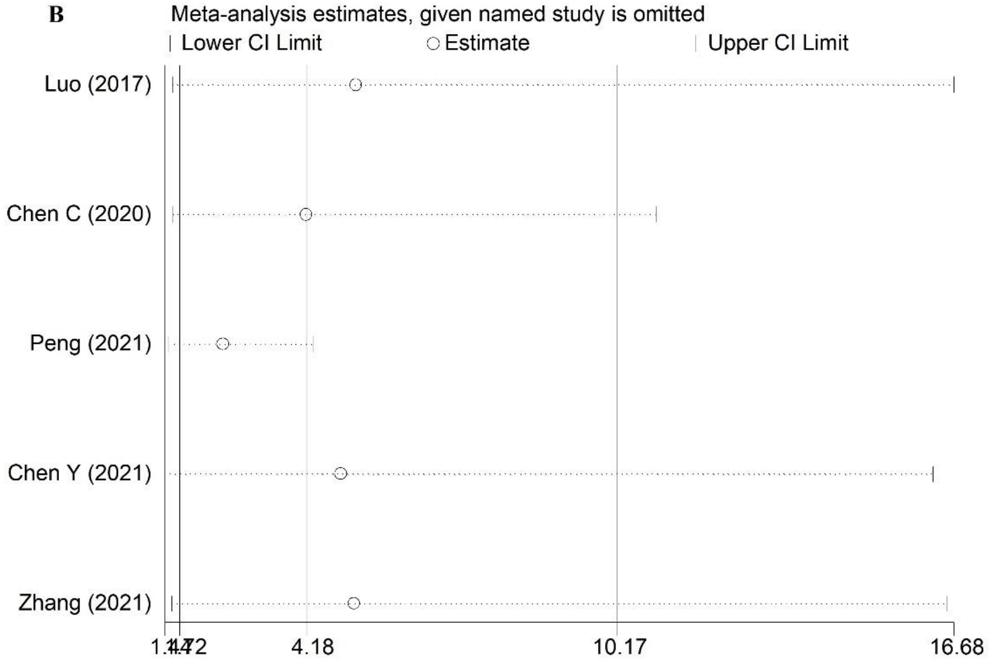
Meta-analysis estimates, given named study is omitted
Lower CI Limit
Estimate
Upper CI Limit
Luo (2017)
Chen C (2020)
Peng (2021)
Chen Y (2021)
Zhang (2021)
4.18
10.17
16.68

C

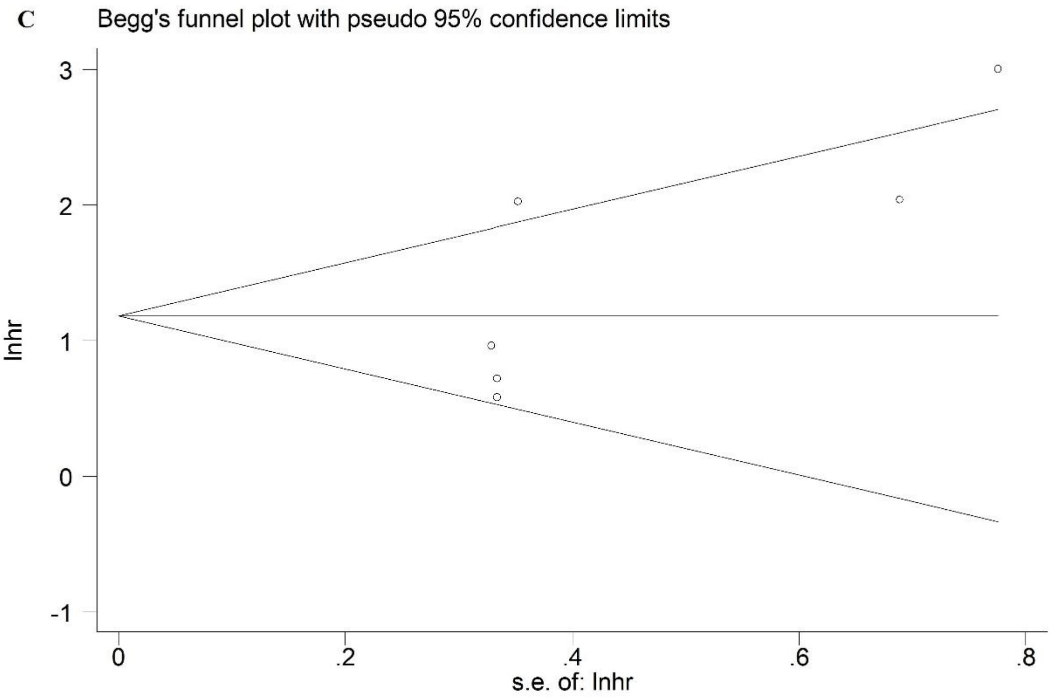

D

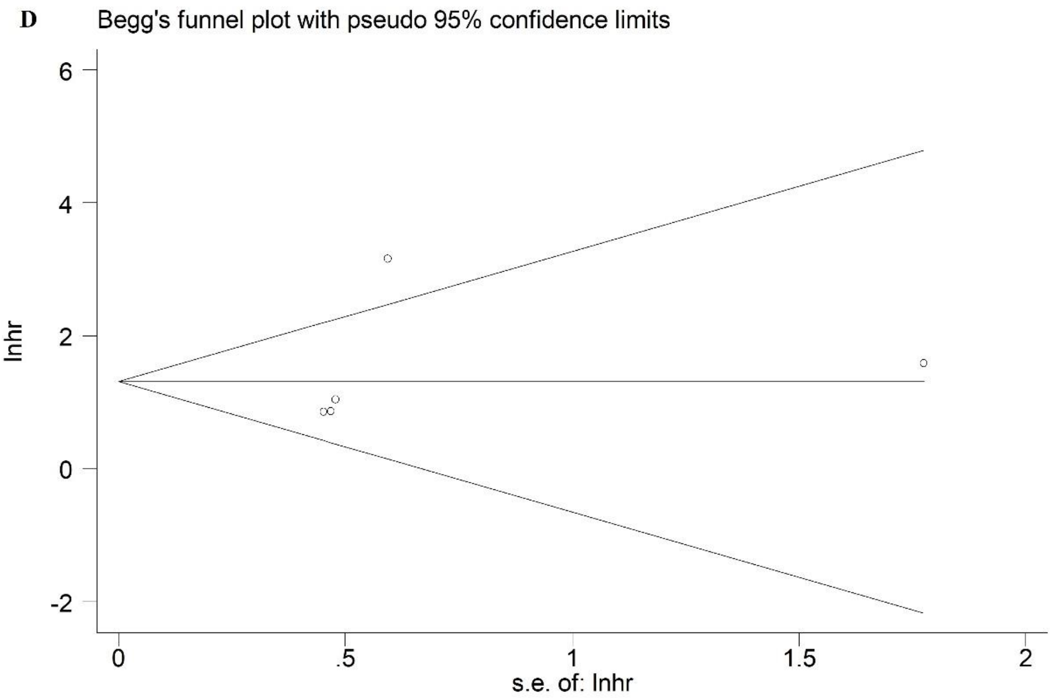

Supplementary figure 3. Sensitivity analysis for the association between solid component and disease-free survival (A) and overall survival (B) in pathological IA stage lung adenocarcinoma patients. Begg’s funnel plot for the association between solid component and disease-free survival (C) and overall survival (D) in pathological IA stage lung adenocarcinoma patients.
